# Stress-Coping and Cortisol Analysis in Patients with Non-Syndromic Cleft Lip and Palate: An Explorative Study

**DOI:** 10.1371/journal.pone.0041015

**Published:** 2012-07-20

**Authors:** Volker Gassling, Paul-Martin Holterhus, Dorothee Herbers, Alexandra Kulle, Uwe Niederberger, Jürgen Hedderich, Jörg Wiltfang, Wolf-Dieter Gerber

**Affiliations:** 1 Department of Oral and Maxillofacial Surgery, University Hospital of Schleswig-Holstein, Kiel, Germany; 2 Department of Pediatrics, Division of Pediatric Endocrinology and Diabetes, University Hospital of Schleswig-Holstein, Kiel, Germany; 3 Institute of Medical Psychology and Medical Sociology, University Hospital of Schleswig-Holstein, Kiel, Germany; 4 Institute of Medical Informatics and Statistics, University Hospital of Schleswig-Holstein, Kiel, Germany; University of Medicine & Dentistry of NJ, United States of America

## Abstract

**Background:**

Non-syndromic clefts of the orofacial region occur in approximately 1 per 500 to 2,500 live births, depending on geographical area and ethnicity. It can be supposed that the disruption of the normal facial structure and the long-standing pressure of treatment from birth to adulthood bring about a range of life stressors which may lead to a long-lasting impact on affected subjects throughout their lives. Therefore, the present study aimed to assess different aspects of psychosocial stress in affected individuals.

**Methods:**

The study was divided into two parts: first, the Trier Social Stress Test which involves uncontrollability and high levels of social-evaluative stress under real conditions and second, the query of various aspects of coping with psychosocial stress. The test group consisted of 30 affected adult subjects, and an equally sized control group of unaffected volunteers. Cortisol dysregulation was determined by saliva samples before and after stress induction. Meanwhile, participants were asked to complete the SVF 120 stress-coping questionnaire.

**Results:**

The analysis of saliva samples showed a similar baseline concentration as well as a similar increase in cortisol levels after stress induction for both groups. Subsequently, the decline in cortisol concentrations was significantly faster in the CLP group (course: p<0.001; groups: p = 0.102; interaction: p = 0.167). The evaluation of the stress-coping questionnaire revealed a significantly shorter rumination about a stressful event in individuals with CLP-related malformations (p = 0.03).

**Conclusion:**

We conclude that adults with CLP have significantly better stress-coping strategies than their healthy peers.

**Trial Registration:**

German Clinical Trials Organization DRKS00003466

## Introduction

**Figure 1:**
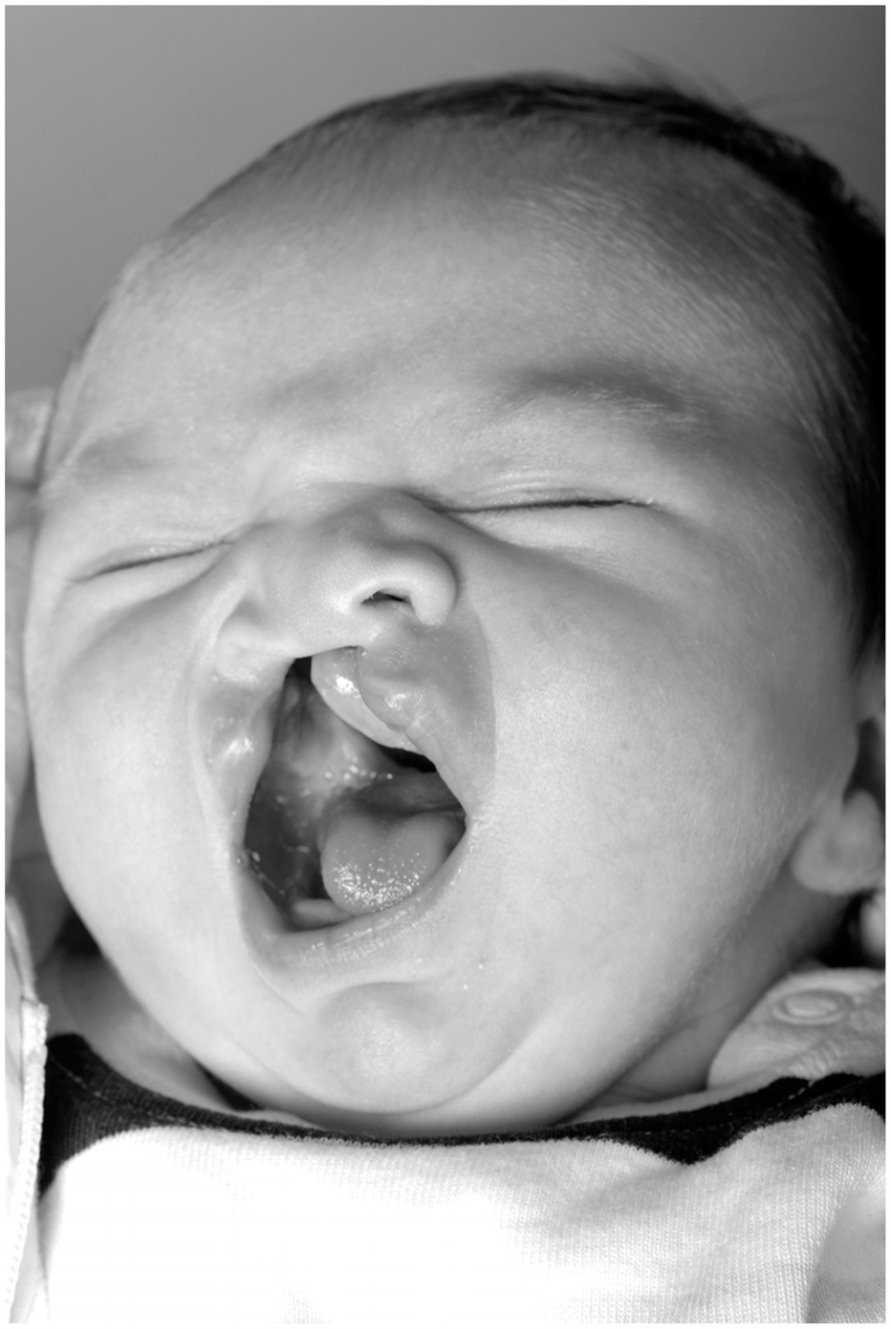
New-born girl aged 1 week with unilateral cleft lip and palate.

Non-syndromic clefts of the orofacial region, including cleft lip, cleft lip and palate or cleft palate alone, occur in approximately 1 per 500 to 2,500 live births, depending on geographical area and ethnicity [1]. The treatment concept of cleft lip and palate (CLP) malformation requires multidisciplinary care, including maxillofacial surgery, orthodontics, otolaryngology, speech therapy, and dentistry. Owing to great efforts, particularly in the field of plastic-surgical treatment during the last few decades, the health outcome of affected patients seems to be good, particularly in developed countries [2]. However, the question is whether the current concept of medical treatment is sufficient or whether there is a need to focus on further aspects which have been neglected in the contemplation of this fateful malformation in the earliest stages of life. It can be supposed that the disruption of the normal facial structure (Figure 1) and the long-standing pressure of treatment over many years have brought about a range of life stressors which may lead to a long-lasting impact on the affected individuals throughout their lives.

In developed countries, psychosocial stress is associated with an increased risk of physical and mental health hazards [3]. Disadvantaged population groups are at particular risk. Factors which enhance this risk are social conditions, recurring pressures and multiple burdens, which lead to chronic stress arousal [4]. Recently, it has been shown that children and adolescents with chronic physical illness show higher levels of depressive symptoms than their unaffected peers, whereby, in addition to chronic fatigue syndrome (d =  .94), and fibromyalgia (d =  .59), the differences were the third strongest in subjects with CLP (d = .54) [5]. Amazingly, it has also been shown that people with CLP have a higher morbidity throughout their lives (from birth up to the age of 55 years) than unaffected individuals. Particularly evident is a significantly increased risk of cancer, cardiovascular diseases, and an increased risk of suicide [6]. Thus, it can be hypothesized that CLP-affected individuals show psychosocial adjustment difficulties which may lead to particular psychosocial stress throughout their lifetimes.

A systematic literature review concerning psychosocial stress and CLP reveals that psychosocial functioning among children and young adults with orofacial clefts is affected by behavioral problems, depression, and unhappiness with their facial appearance and speech [7]. More precisely, dissatisfaction with facial appearance could be localized to the upper lip, and the nose and nasal breathing. Furthermore, even a positive correlation between satisfaction with facial appearance and a health-related quality of life has been found in adults with bilateral CLP [8]. Several years ago, it was shown that the severity of the cleft deformity may have a significant impact on social competence in childhood, e.g. in the development of friendships. From this, it is not surprising that even adult siblings with repaired clefts were shown to be less likely to marry than their non-cleft siblings [9]. Recently, these findings have been explained by a comparison of a visual assessment of faces and it was found that in CLP faces there were more initial fixations in the mouth and longer fixations in the mouth and nose regions compared to control faces. In addition, CLP faces were rated more negatively overall [10]. Noteworthy is however that a further investigation into psychosocial adjustment difficulties in adolescents with orofacial clefts revealed greater self-satisfaction with several aspects of facial appearance (e.g. teeth, eyes, ears, hair, and chin) compared with adolescents without facial disfigurement. However, there was dissatisfaction with lip and nose appearance compared to the control group. Nevertheless, mothers were significantly more satisfied with the facial appearance of their adolescents than with themselves [11].

As mentioned above, even speech problems have been found to affect the psychosocial adjustment of individuals with clefts of the orofacial region. Recently, these findings have been confirmed in adolescents who have reported speech problems as an important factor in psychosocial adjustment difficulties [12]. Amazingly, it has been found that speech problems, and not more or less abnormal facial appearance, lead to better psychosocial health in adolescents [13]. On the other hand, it is known that an important indicator of psychosocial adjustment difficulties is the occurrence of teasing in childhood. In this regard, several studies have pointed out a positive correlation between the conspicuous lip appearance and speech problems [14], [15]. Worthy of mention here is that the orofacial cleft per se was rated less seriously than teasing in the past [7]. To summarize the above-mentioned findings, it can be assumed that concerns about facial appearance and speech are the major features of psychosocial adjustment difficulties in CLP-related malformations.

From a definition of coping going back to Folkman and Lazarus, positive coping strategies have been shown to reduce health-related risk factors in general [16]. The theory of transactional stress-coping suggested by Folkman and Lazarus is directed towards three essential aspects of appraisal [17], [18]. The primary appraisal indicates the skill to recognize a stressful event, the secondary appraisal is the skill to have the appropriate strategies to cope, and the third appraisal is directed towards a new evaluation of a stressful event.

The current study aimed at examining different aspects of psychosocial stress in adults with a cleft of the orofacial region. In the first part, focus was given to the question of whether adults with CLP-related malformations and their healthy peers showed different kinds of hypothalamic-pituitary-adrenal (HPA) axis alterations, i.e. cortisol dysregulation, during a standardized stress situation under real conditions (Trier Social Stress Test (TSST)) by screening cortisol levels in saliva. For saliva samples, the TSST is the most used stress test in stress induction research which measures cortisol simultaneously. The test has been shown to be a valid model for the experimental induction of stress (independent variable) and cortisol (dependent variable) [19]. In the second part, various aspects of psychosocial stress were investigated using a standardized stress-coping questionnaire.

**Figure 2:**
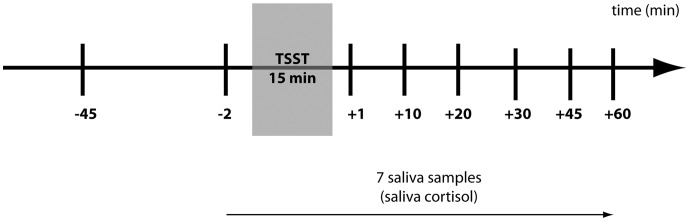
Sample acquisition in the course of the Trier Social Stress Test (TSST).

## Materials and Methods

The protocol for this trial and supporting CONSORT checklist are available as supporting information; see Checklist S1 and Protocol S1.

### Ethics Statement

All participating subjects were recruited from consultation hours for craniofacial malformations at the Department of Cranio-Maxillofacial Surgery, University Hospital Schleswig-Holstein, Campus Kiel. They were informed about the course of the experimental procedure and gave their written informed consent according to the Helsinki convention before examination began. The study was approved by the ethics board of the Christian-Albrechts-University of Kiel, Germany (Ref: D 433/10), and it was registered in the German Clinical Trials Register (registration code DRKS00003466).

### Study Design

#### Part I - Trier social stress test

The TSST, first described by Kirschbaum et al. [20], was designed for the induction of moderate psychosocial stress under laboratory conditions. In brief, each participant received the task of playing the role of a candidate during a mock job interview in front of an audience. They were told that particular attention would be given to behavior, gestures, mimicry, and voice and that they would be filmed for further analysis. Furthermore, the test person had to perform a difficult mental arithmetic exercise out loud. Saliva samples were taken for later determination of cortisol levels at different time points before and after the stress induction. A general schematic overview of sample acquisition is given in Figure 2. One important precondition for the TSST is that the test procedure is completely standardized and sufficient reliability and validity have been shown in several studies.

#### Sample acquisition

Samples were collected using Salivette® collection devices (Sarstedt, Nümbrecht, Germany), and stored at −20°C until biochemical analysis. In particular, uncentrifuged samples are stable for at least three months [21].

#### Salivary cortisol analysis

Cortisol was analyzed by ultra-performance liquid chromatography coupled with tandem mass spectrometry (UPLC-MS/MS) (Waters, Milford, MA, USA) according to previously described methods [22]. The method was validated for saliva analysis. In brief, aliquots of samples, calibrators and controls with a volume of 0.1 ml were mixed with an internal standard. Deuterium-labeled cortisol was used as the internal standard, i.e. cortisol-d4. Calibrators and controls were prepared in steroid-free saliva. Steroid-free saliva was generated by stirring saliva from volunteers three times with active charcoal and subsequent filtration. All samples were extracted using Oasis MAX SPE system plates (Waters, Milford, MA, USA). The UPLC-MS/MS system was used in the multiple reaction mode (MRM), hormones were measured using an ESI probe in the positive ion mode. For cortisol, two different MRM transitions were monitored m/z 363.6/121.2 and m/z 363.3/97.2. The limit of detection (LOD) was determined as the lowest concentration of cortisol at which the chromatogram was present in both transitions at the excepted retention time with a signal at least three times higher than the baseline noise. The limit of quantification (LOQ) was determined by analyzing seven samples in duplicate containing progressively, equidistantly lower concentrations of steroid hormones down to 0.001 nmol/L. For the cortisol, the LOD was 0.05 nmol/L and the LOQ was 0.1 nmol/L determined in case of imprecision of CV >20%. The sensitivity of the assay, within runs (intra-assay) and between runs (inter-assay), as well as the coefficient of variation (% CV) for replicate quality controls averaged for different concentrations of cortisol were between 0.7% and 6.7% (supplemental data). The total run time for the cortisol assay was 3 min.

#### Part II – Stress-coping questionnaire (SVF 120)

Cognitive and behavior-orientated management measures were assessed using the SVF 120 stress-coping questionnaire. The test was performed according to the method first described by Janke et al. [23]. Each participant has to complete the questionnaire mentioned above after the TSST.

### Data Transformation and Statistical Analysis

Due to better approximation of normal distribution, cortisol data were transformed before statistical analyses using natural logarithm following the formula cortisol value ln  =  ln (cortisol value +1) [24]–[27].

Statistical methods were intended to evaluate important aspects in stress-coping parameters in an exploratory approach. SPSS for Windows, Version 18, was used for all statistical analyses, including descriptive statistics. Prior to all analyses, univariate and multivariate normal distributions were tested by Kolmogorov-Smirnov goodness-of-fit tests resp. multivariate box-tests for each variable resp. set of variables. Repeated-measures analyses of variance (ANOVA) with group (CLP patients, controls) as the between-subject factor, and course (cortisol profile: m1– m7) as the within-subject factor were computed for the cortisol response. T-tests for independent groups for the psychometric scales were also applied. Comparisons of distributions were done by Chi^2^-test. Finally, Pearson product-moment correlations between cortisol response and the psychometric scales were computed. P-values are stated without any adjustments in an exploratory way. The level of significance was set to p≤0.05.

**Table 1:** Demographic characteristics of the CLP group (n = 30) and the control group (n = 30).

	CLP Group	Control Group	
**Age (years)**			
Mean	27.2	27.8	p = 0.428
Sd	8.3	9.6	
min-max	18–49	18–51	
**Gender**			
W	14 (46.7%)	15 (50%)	p>0.999
M	16 (53.3%)	15 (50%)	

sd = standard deviation; min-max = minimum and maximum; w = women; m = men.

## Results

### Age and Gender of Participants

In course of the recruitment, 250 patients were contacted by letter, whereby 47 subjects declined participation, and 32 subjects gave their commitment. The test and control groups had similar demographic characteristics. Thirty adult patients with a unilateral cleft of the lip and palate (mean age 27.2 years, SD 8.3) were enclosed in the test group. The control group consisting of 30 unaffected volunteers (mean age 27.8 years, SD 9.6) from Schleswig-Holstein, who were recruited by newspaper advertisements. The minimum and maximum ages were 18 and 49 years in the CLP group and 18 and 51 years in the control group, respectively (p = 0.428). The CLP group contained 14 (46.7%) females and 16 (53.3%) males. There were 15 males and females each (p>0.999) in the control group (Table 1). None of the subjects of either group reported mental or psychiatric problems by psychiatric interviews.

### Trier Social Stress Test

**Figure 3:**
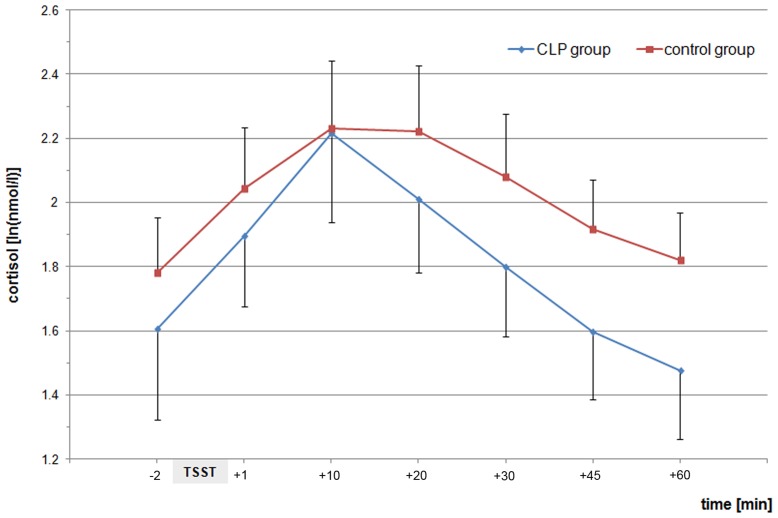
Mean saliva cortisol concentrations [ln(nmol/l)] (± standard deviation) at different time points (min) for the CLP group and the control group during the Trier Social Stress Test (TSST).

The analysis of the collected saliva samples showed approximately equal cortisol levels at baseline for the CLP and the control group, respectively. After stress induction there was a similar increase in cortisol concentrations in both groups. Subsequently, the decline in cortisol concentrations was significantly faster in the CLP group (course: p<0.001; groups: p = 0.102; interaction: p = 0.167). In particular, significant differences in cortisol concentrations were be detected between groups at 45^th^ and 60^th^ minute (t45: p = 0.018; t60: p = 0.011) (Table 2, Figure 3).

**Table 2:** Course of log cortisol concentrations [ln(nmol/l)] at different time points (min) for the CLP group and the control group during the Trier Social Stress Test (TSST).

	CLP Group		Control Group		
Time	m	sd	m	sd	p
− 2	1.607	0.780	1.780	0.475	0.305
+1	1.895	0.598	2.043	0.526	0.314
+10	2.217	0.762	2.231	0.576	0.937
+20	2.012	0.635	2.222	0.564	0.182
+30	1.800	0.598	2.081	0.533	0.060
+45	1.598	0.578	1.917	0.419	0.018
+60	1.477	0.585	1.819	0.405	0.011

m = mean; sd = standard deviations; p = p-values from t-test.

### Stress-coping Questionnaire (SVF 120)

The evaluation of the SVF 120 which asks about 19 separate stress-coping strategies showed significant differences (p = 0.03) for ruminating about a stressful event for both groups. It was shown that adults with facial disfigurement in their medical history showed a significant shorter rumination about an acutely stressful event than the unaffected control group (Table 3).

**Table 3:** Stress-coping questionnaire (SVF 120); 19 different subscales for stress-coping in the CLP group and the control group.

	CLP Group		Control Group		
Subscales	m	sd	m	sd	p
trivialisation/minimalisation	13.1	3.9	11.6	4.1	0.14
de-emphasis by comparison with others	11.4	4.3	9.4	4.6	0.09
rejection of guilt	11.1	3.1	9.8	4.0	0.17
distraction/deflection from a situation	14.0	4.0	13.1	3.8	0.38
vicarious satisfaction	11.0	4.5	10.1	4.4	0.45
search for self-affirmation	12.5	4.1	11.1	4.6	0.22
attempt to control situation	15.7	4.8	17.4	4.2	0.14
response control	14.8	4.1	14.5	3.4	0.76
positive self-instruction	16.3	4.3	15.1	4.2	0.28
need for social support	12.0	5.5	14.6	5.7	0.08
avoidance tendencies	11.6	4.7	11.9	3.3	0.08
escapist tendencies	10.6	4.5	10.8	4.9	0.87
social isolation	8.2	5.9	8.1	5.1	0.98
mental perseveration	12.3	5.5	16.5	4.9	0.03
resignation	7.0	4.8	8.2	4.8	0.32
self-pity	9.2	4.8	8.5	4.2	0.53
self-incrimination	10.0	4.6	10.5	4.1	0.62
aggression	10.0	4.6	8.2	4.4	0.12
medicine-taking	1.9	2.9	1.9	2.6	0.93

m = mean; sd = standard deviation; p = p-values from t-test.

## Discussion

The current explorative study aimed at examining different aspects of psychosocial stress in adults with a cleft of the orofacial region. There were two findings of particular interest. First, the analysis of saliva samples showed approximately equal cortisol levels at baseline and a similar increase in cortisol concentrations for the CLP and the control group, respectively. Subsequently, the decline in cortisol concentrations was significantly faster in the CLP group. Second, the evaluation of a SVF 120 showed significant differences for the subscale ‘ruminating about a stressful event’ for both groups. In particular, adults with CLP showed a significant shorter rumination about an acutely stressful event then the control group.

Children born with a facial disfigurement are forced to undergo multiple surgical procedures and frequent clinic attendances from birth to adulthood. The result is a more or less intensive disturbance of facial appearance and speech impediment which might lead to particular psychosocial stress in affected individuals.

Besides acute stress, chronic stress is associated with increased risk of mental and physical health hazards [3]. It has been shown that chronic stress with its ongoing activation of the HPA axis can lead to cortisol dysregulation and thus is thought to result in several kinds of negative health outcomes [28]. An essential prerequisite for testing the HPA axis function is the assessment of the endogenous response of the HPA axis to mental stress [29], whereby the determination of cortisol is supposed to be a suitable method [30]. In particular, salivary cortisol is commonly accepted as an appropriate bioindicator [31]. The investigation of stress, both physical and mental, requires an appropriate test method. When the present study was being planned, the scientific essence of the above-mentioned considerations were taken into account to ensure that the main suspected issues of psychosocial stress in CLP-related malformations, i.e. facial appearance and speech, were sought. These limitations led to the TSST, which accurately simulates the main stressors speech and facial appearance by a mock interview. Furthermore, the test guaranteed two essential test properties, i.e. uncontrollability and high levels of social-evaluative threat, under real conditions paired with largest cortisol changes and the longest times to recovery [32]. Until now, it has been widely used to stimulate psychosocial stress under laboratory conditions [33], [34].

Here, for the very first time, psychosocial stress was investigated in CLP-related malformation by the widely accepted method of TSST. The overall values of cortisol found in the present study were three times lower than in other publications [35]. This is caused by the different measuring methods used. It is known that the cortisol concentration in saliva is directly proportional to the free serum unbound cortisol in blood [36]. Usually, unbound or free cortisol in plasma is measured by enzyme-linked immunosorbent assay (ELISA) or radio-immunoassay (RIA). Both immunological methods are sensitive but they have a number of limitations, mostly related to the lack of specificity due to cross-reactivity of antibodies, which leads to an over- or underestimated true cortisol concentration. Furthermore, differences in techniques used in different laboratories impede comparability [37]. Therefore, a more accurate and precise detection method of cortisol is needed. Liquid chromatography-tandem mass spectrometry (LC-MS/MS) has become the method of choice for clinical steroid analysis in the last few years. Several LC-MS/MS methods for determination of cortisol in saliva have been developed [38]–[40]. The present study used an UPLC-MS/MS assay for the analysis of saliva cortisol. A further important aspect and an essential advantage for the determination of cortisol levels in saliva is the painless specimen collection and hence the absence of additional pain-related HPA axis alteration in the course of sample acquisition.

Furthermore, the interpretation of the present findings was rather difficult due to the gap in the international literature concerning psychosocial stress and cortisol alterations in CLP-related conditions. Most publications on psychosocial stress and cortisol alterations deal with mental illness and thus the question arises as to whether these data are comparable with data received from CLP-related malformations at all. This was deemed not to be the case and so was excluded from the discussion.

However, it can be supposed that CLP-affected individuals with their particular psychosocial adjustment difficulties, i.e. facial appearance and speech, may have a vulnerable endocrinological stress axis. It is remarkable that against our expectations cortisol levels were at the same magnitude for the baseline and up to the 10^th^ minute after stress induction (p = 0.937). Subsequently, from the 45^th^ minute, the decline in cortisol levels was significantly greater in the CLP group than in the control group. A possible explanation for these findings in general was given some years ago by Dickerson & Kemeny [32] who pointed out that: ‘Indeed, a quick, strong HPA response coupled with rapid recovery in many cases would be adaptive, providing the organism with the necessary energy to reduce goal threat’. It seems as if individuals with CLP have such a particular endocrinological stress response, maybe simply because of their stigmatization. It is conceivable that the lives of CLP-affected subjects, with their special challenges and burdens, result in resilience [41]. In general, it is supposed that individuals with major life stressors in their history can benefit in the sense of positive outcomes [42]. In this context, it has been shown that children with CLP showed good resilience with adequate emotional functioning, high satisfaction with appearance, and lower frequency of reported teasing. In contrast, it has been shown that the child’s characteristics such as visibility of cleft, gender, and additional diagnosis do not generally contribute to the explanation of psychosocial resilience [43]. The knowledge of resilience is still in flux and it is now believed that influencing factors belong to a dynamic model with multiple levels of interaction, including research into the neurobiology of stress and adaption, epigenetic processes, and disasters [44]. Thus, it is difficult to assess all the factors involved. However, it is known that a major topic of resilience in childhood is the perceived social support. Thus, it can be suggested that the present results in part are caused by the particular social support from early life time to adulthood which may protect humans with CLP against adverse life events. Recently, it has been shown that subjects with malformations, i.e. CLP, receive more social support from parents and friends in their lifetime, resulting only in a small degree of negative outcomes (e.g. psychological distress) [45]. These findings were given in a publication which illuminates the parent’s perspective. A comparison of children with orofacial clefts with children suffering from other disabilities showed less parental stress if children suffer from additional behavioral disorders [46].

In this context, the present findings of the stress-coping questionnaire could be interpreted. The subscale rumination or mental preservation showed a significantly shorter rumination about a stressful event for the CLP group compared with the control group. Some years ago it was shown that rumination in general leads to prolonged cortisol activation, i.e. delayed recovery from a psychosocial stressor [47]. Recently, these findings could be confirmed by Zoccola et al., who found that trait-related ruminating led to a greater reactivity of cortisol secretion as well as delayed recovery in response to an acute laboratory psychosocial stressor (modified Trier Social Stress Test) [34]. Thus, it can be hypothesized that the significantly shorter rumination in the CLP group is associated with a significantly faster recovery of cortisol levels after stress induction in the course of the TSST. In conclusion, our findings are in accordance with findings that show that HPA axis dysregulation can be reduced by cognitive-behavioral stress management training [48].

## Supporting Information

Checklist S1 **CONSORT Checklist.**
(DOC)

Protocol S1 **Trial Protocol.**
(DOCX)
