# Supplementary material for: Stress-Coping and Cortisol Analysis in Patients with Non-Syndromic Cleft Lip and Palate: An Explorative Study
Source: PLoS One. 2012 Jul 20;7(7):e41015. doi: 10.1371/journal.pone.0041015 (PMC3401206; doi:10.1371/journal.pone.0041015)
Supplement: Protocol S1 — Trial Protocol. (DOCX) [file pone.0041015.s002.docx]

The Trier Social Stress Test (TSST)

iss

dhd

**A Laboratory Stress Protocol**

**Introduction**

The Trier Social Stress Test (TSST) was developed at the University of Trier, Germany. It has become a standard protocol for the experimental induction of moderate psychological stress in psychobiological research. In the past ten years, more than 4000 TSST sessions have been performed worldwide (Kudielka et al., 2007). We have been using the TSST for several years for induction of moderate psychosocial stress under laboratory conditions with special emphasis on changes in hypothalamic-pituitary adrenal (HPA) axis activity, one of the organism’s major physiological stress systems. This stress protocol has been found to induce significant cortisol, ACTH, and cardiovascular responses at first exposure in 70-80% of all subjects. In a recent meta-analysis, protocols combining uncontrollability and social-evaluative threats - such as the TSST - have been found to produce the largest HPA axis responses and have been associated with the longest recovery times (Dickerson et al., 2004).

To ensure that the results obtained in this test remain comparable over time, it is necessary to standardize its execution, so that every research participant is challenged similarly during confrontation with the TSST. Therefore, all persons involved in the TSST (pre-TSST experimenter, TSST, post-TSST experimenter, see below) should be familiar with the exact procedure of this stress protocol.

# General Protocol

Each research participant spends about two hours and 30 minutes in the laboratory. This time frame consists of a 45 minutes of resting phase prior to the TSST (if blood is taken), 15 minutes of psychosocial stress (TSST) and 90 minutes of resting phase for post-test assessments and debriefing.

Three experimenters are involved: two panel members conduct the actual TSST and one experimenter overlooks the whole protocol.

## Reception

On arrival, the research participant is welcomed outside the experiment rooms by the experimenter responsible for the participants’ reception and (subsequent) introduction to the TSST. They are then led to Experiment Room 1, which serves as the rest area.

## Resting Phase and First Saliva Samples

45 minutes of resting phase is now necessary to allow possible HPA axis and sympathetic nervous system activation to return to baseline levels. (If blood samples are to be obtained, a venous catheter is inserted at this time.)

Two minutes prior to the TSST, the first saliva (and blood) samples are taken.

If the study design requires pre-TSST assessment, questionnaires can be filled out by the participant during the resting phase.

In addition, the heart rate can be assessed continuously using a wireless signal transmission device, which is administered 20 minutes prior to the TSST.

## Pre-TSST: Introduction

The research participant is then led to Experiment Room 2.

This room is set up in the following way: two people are seated behind a table in front of the wall facing the door. A microphone is put up in the middle of the room and a video camera is directed towards it. On the table, there is a recording device, which is connected to the microphone and a data protocol sheet is put in front of each panel member. The experimenter explains to the research participants their first task, which is to give an introductory talk in front of the panel present, in which he/she is to imagine having been invited to an interview to apply for a job. (experimenter introductions: see below)

## TSST: Preparation Phase

The experimenter than leaves and the participant stays in Experiment Room 2, were he/she has three minutes to prepare his/her talk. The participant is allowed to take notes but he/she must not use them during his/her speech in front of the audience.

## TSST: Task 1 (free speech)

The camera and recorder are switched on and the research participant is asked to step in front of the microphone and start the talk.

All members of the audience remain quiet, as long as the research participant continues to speak fluently. Only after a pause of more than twenty seconds prior to the end of the five-minute period are questions asked: these are e.g.*"What qualifies you in particular for this position?"* (see below)

## TSST: Task 2 (arithmetic task)

After the five-minute period the research participant is then informed by the chair about the second half of his/her task (instructions: see below). This part of the test should be concluded after a maximum of five minutes, insofar as the participant does not reach a count of "0" before that. It is recommended that prime numbers be used as subtractors for this task, since these make the task more difficult.

## Post-TSST: Further Assessment (saliva samples and questionnaires)

The research participants are then led back to Experiment Room 1, where the post-test assessment takes place. This phase consists of the collection of additional saliva (and blood) samples (+1, +10, +20, +30, +45, +60, +90 minutes relative to the end of TSST) and the filling out of questionnaires.

## Debriefing

If there is only a single occurrence of the TSST in the course of the experiment, the experimenter can give information about the purpose of the study, i.e. by "debriefing" the research participant after the last saliva (and blood) sample has been taken. This step, however, is not recommended if additional run-throughs of the TSST with this participant are planned. In this case, the ‘post-TSST experimenter’ can refer to later explanations about the experiment and the goals of the study. On the last day, there should always be a complete explanation; it is also helpful if the panel members participate in these explanations.

**TSST Course**


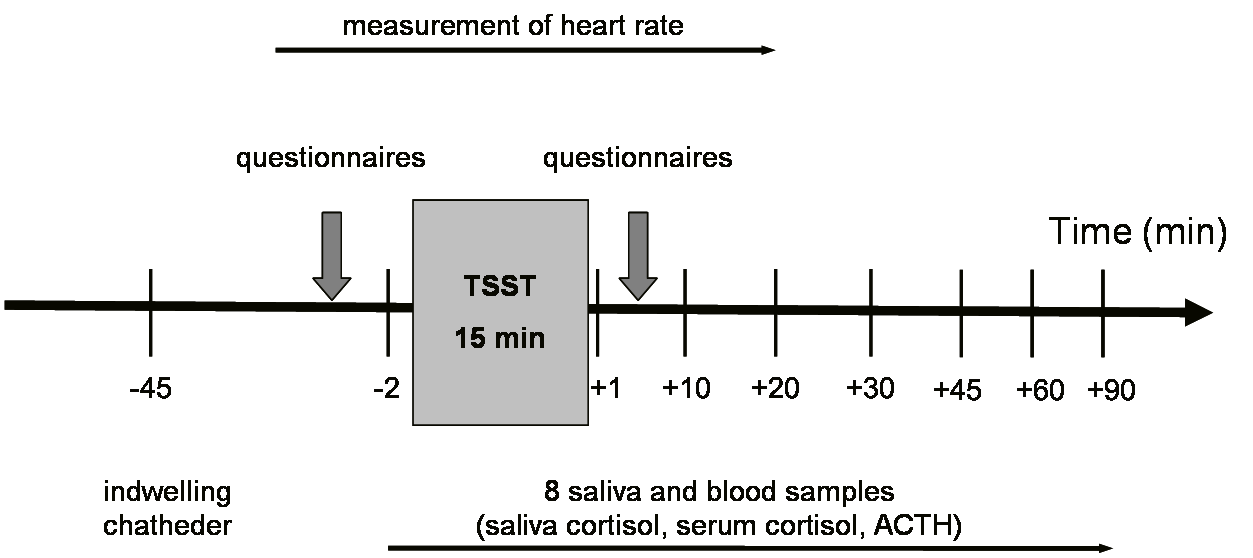


Figure 1: TSST Course

#
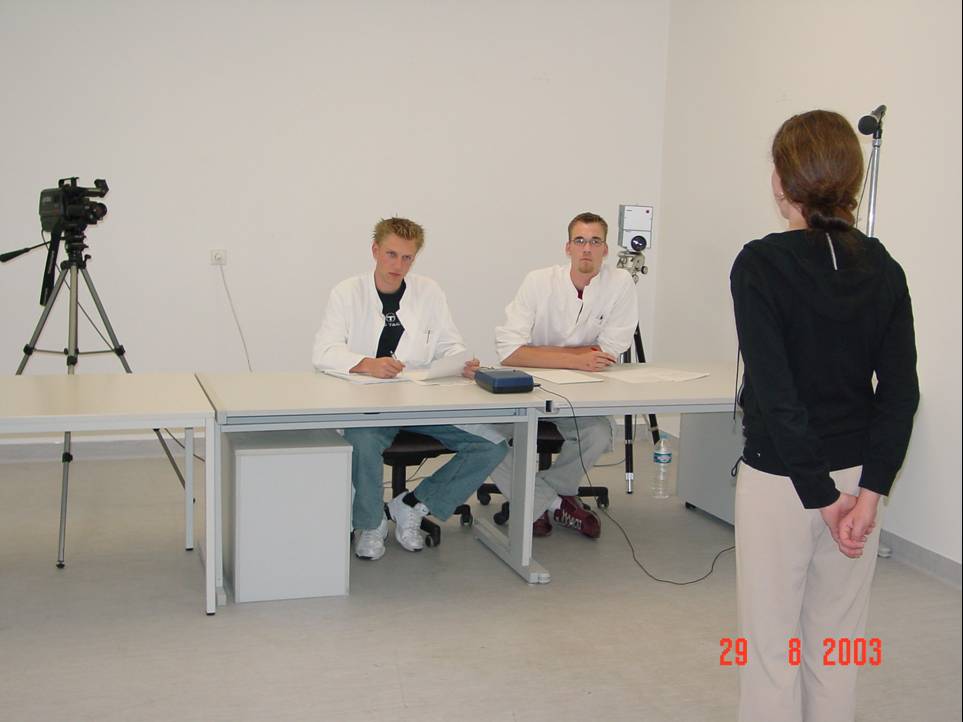


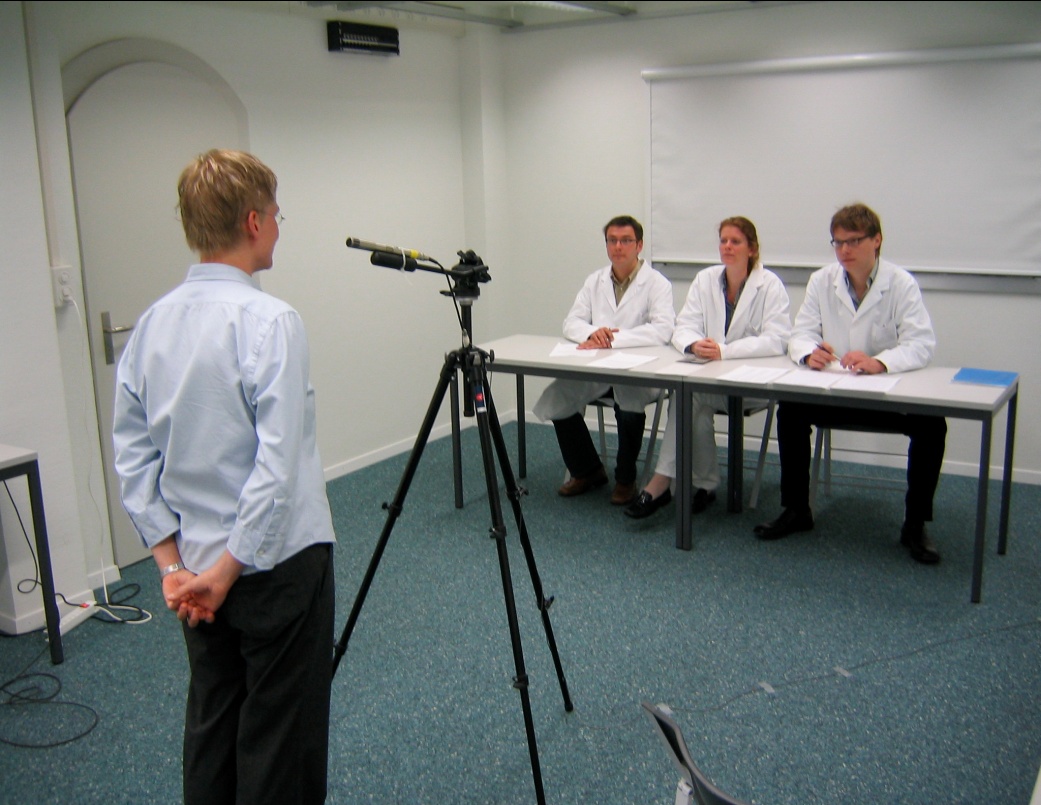


Figure 2: Experiment Room 2

# Tasks for the different experimenters

## The Experimenter

### Timing

The experimenter who welcomes the research participants is responsible for the instruction and timely coordination of the research participant. Since the exact schedule of the collection of saliva (and blood) samples is essential in order to compare data, it is recommended that the pre- (and post-) TSST experimenter deploy a stopwatch if he/she should lack experience.

### Check adherence to participation conditions

When receiving the research participant, it is important to ask about health-related limitations or possible stimulation of the HPA. Even though the participants will have been informed about participation conditions at the time of scheduling the appointment, this is still a good opportunity to double-check adherence to these conditions. One should ask at this point about illnesses, physical activities just prior to arrival, cigarettes smoked, and medications taken. In case of doubt: take notes and ask later; what is documented can be checked subsequently. Depending on the specific goal of the study, there are different exclusion criteria, of which the experimenter should be informed (e.g. hormone treatment).

### Objectivity

Before and during the rest period the research participant should not be informed in great detail about the procedure of the experiment; if the participant can exactly anticipate what comes his/her way, this in itself could have a stimulating effect on the HPA axis. If there should be any questions about the specific content of the experiment, the experimenter should answer with a general statement about a psychological stress-situation and refer to a subsequent introduction for details. After the reception, the participant should be led to the rest area; at this point the purpose of the rest can be explained. Note the exact time of the beginning of the rest period.

### Introduction to the TSST

The experimenter leads the participant to Experiment Room 2 and introduces him/her to the first TSST task by saying *"Your task in this experiment is the following: please imagine that you have applied for a job and have been invited for an interview. In contrast to a real interview, however, you are supposed to give a talk, in which you are to convince the panel in five minutes why you think that you would be the best candidate for this position. Please note that you will be recorded by a camera and a microphone for subsequent voice and behavioural analysis. The members are trained in behavioural analysis and will take notes during your talk. You should try to leave the best possible impression, and assume the role of the applicant for the duration of the talk as best as you can. The panel will reserve the right to ask follow-up questions in case of uncertainties to receive all necessary information from you. Following your talk, you will be given a second task by the panel, which will only be explained to you by the panel. You may take some notes now, which you must not use during your talk. Do you have any questions?"*

## 'The TSST Panel Members'

### Neutral impression

A crucial characteristic of the TSST is the impression that the panel should make. The principal aspect of the TSST is the role play, and for that it is important that everybody involved play their respective roles to the best of their abilities. As for the panel, which has to decide about the acceptance of an applicant for a specific position, the issue is therefore to make an impression that leaves no doubt about the seriousness of this endeavour. Furthermore, the TSST is meant to be a psychological stress situation; for that, as well, it is important to maintain a serious impression.

In any case, talk about the situation as such should be avoided. Any role play loses its realism (and with that its stress-inducing effect) if it is made research participant of a discussion. It is clear that a real job interview would never take place like this in real life and that the TSST can only be a compromise - however, that should only, if at all, be discussed at the time of the introduction or post-test assessments and debriefing, but not during the actual task. Therefore, it is recommended that during the introduction by the experimenter none of the panel members talk or smile; should the research participant address the panel, one should only return the greeting courteously. If necessary, it can be pointed out that any questions of the research participant should be directed to the experimenter, rather than to the panel.

Furthermore, all panel members should seek eye contact with the participant during the talk; the knowledge that all persons present give the research participant their undivided attention further reinforces the seriousness of the situation for the research participant. Of course, there should be no laughing during the talk.

Much has been said about psychological stress tests, therefore only a short remark at this point: the point of these questions is not to embarrass the research participants or to be mean to him/her. This is neither the purpose nor the task of the TSST and would also distort the contents of this role play. The research participant’s task is to present him/herself before an audience. The questions should serve to deepen this presentation and to receive information about specific qualities of the applicant. This is all. Questions such as "Do you have friends?" are not called for in this context.

### Taking notes

The actual task of the panel starts three minutes after the research participant has begun taking notes.

(Furthermore, the panel can note the number of errors and the number that the research participant has eventually reached as a performance measure)

## The TSST Chairperson of the Panel

### Opening of the session, starting of taping devices

At this time, the chairperson of the panel should turn on the video camera by hand or remote control (making sure he/she knows the operating instructions beforehand). He/she opens up the session with the words “*Please step behind the line and begin your talk"*. Simultaneously, the tape recorder is turned on; although only a decoy, this gesture is of great importance in light of the situational context.

### Addressing the research participant for Task 1

Only the chairperson should address the research participant directly, so that coordination problems between the panel members can be avoided.

The chair should let the research participant speak for the first three minutes. In most cases, the participant will come to the end of the talk even before three minutes have passed. The chair should then give him/her time to formulate additional elaborations. In any case, there should be a pause.

After about a twenty-second pause, the chair can alert the research participant to the remaining time, as with the phrase *"You still have time, please continue...".* Should it appear after another ten seconds that the participant has nothing further to say, then the chair should ask questions until the end of the time period. The phrasing of these questions is left to the chair's discretion; it may also be solely oriented towards the participants’ previous statements.

Typical questions in this context are:

- *Why do you think you are especially well-qualified for this task?*
- *Why do you think you are better qualified then the other applicants?*
- *What do your family/ friends especially appreciate about you?*
- *What do you appreciate about your friends?*
- *What do you appreciate about colleagues?*

(further questions: see ‘Material’)

Only in rare instances, will the research participant be able to talk alone for the full five minutes. In this case, it is left to the discretion of the chair whether he/she wants to intervene between the third and fifth minute to ask questions of the research participant or whether the participant is allowed to continue. This should also be dependent on what is being said by the participant. For instance, it is not appropriate for the applicant to speak in great detail about specific lessons he/she may have learned in the course of training at university or elsewhere. Some research participants use their school-knowledge to distract from their own person. In this case, the chair should certainly intervene, for example by saying *"We believe you that you know how to execute a market analysis, but we would be more interested to find out why you were so involved in or drawn to this area*."

### Addressing the research participant for Task 2

After the first five minutes, it is the chair's duty to explain the second part of the stress protocol. To avoid the possibility of the research participant becoming annoyed, it is very important to make it clear that this is indeed a second task that has nothing to do with the application talk. In the past, some participants have refused to engage in mental arithmetic because they felt (rightly so) that it had nothing to do with their job application.

A typical transition would sound like this:

*“We now want you to solve a calculation task. Please count aloud backwards from 2023 to zero in 17-step sequences. Please calculate as quickly and correctly as possible. Should you miscalculate, we will point out your mistake and you have to start all over again. Do you have any questions?"*

Protocol sheets with all intermediate numbers are available in the panel room (see ‘Material‘); the members of the panel do therefore not have to calculate themselves.

Should the research participant miscalculate, the chair will respond with the standard phrase "*Error. 2023*." until the end of the test period.

### End of the session

At the end of the test period, the chair should thank the research participant for his/her participation and ask him/her to go to the neighbouring room for post-test assessments and debriefing. With that, the panel's role in the TSST is concluded.

**‘The Post-TSST Experimenter’**

### Saliva samples

Post-test assessments and debriefing are also conducted by the experimenter. It is very important to adhere to the time of cortisol sampling. For this purpose, the experimenter notes the exact time when the participant enters the post-test assessments and debriefing room.

### Further assessment e.g. questionnaires

In between sampling periods, the research participant has an opportunity to fill out questionnaires, insofar as they are needed (depending on the experimental design).

# Important References

Dickerson, S. S., Kemeny, M. E. (2004). Acute stressors and cortisol responses: a theoretical integration and synthesis of laboratory research*.* Psychological Bulletin, 130(3), 355-91.

Kirschbaum, C., Pirke, K. M., Hellhammer, D. H. (1993). The 'Trier Social Stress Test' - A tool for investigating psychobiological stress responses in a laboratory setting. Neuropsychobiology, 28(1-2), 76-81.

Kudielka, B. M., Hellhammer, D. H., Kirschbaum, C. (2007). Ten years of research with the Trier Social Stress Test (TSST) - revisited. In E. Harmon-Jones, P. Winkelman (Eds.). Social Neuroscience: Integrating biological and psychological explanations of behavior (pp.56-83). New York: Guilford Press.

# Material

## The ‘Pre-TSST Experimenters’ Introduction

"Your task in this experiment is the following: please imagine that you have applied for a job and have been invited for an interview. In contrast to a real interview, however, you are supposed to give a talk, in which you are to convince the panel in five minutes why you think that you would be the best candidate for this position. Please note that you will be recorded by a camera and a microphone for subsequent voice and behavioural analysis. The panel members are trained in behavioural analysis and will take notes during your talk. You should try to leave the best possible impression, and assume the role of the applicant for the duration of the talk as best as you can. The panel will reserve the right to ask follow-up questions in case of uncertainties to receive all necessary information from you. Following your talk, you will be given a second task by the panel, which will only be explained to you by the panel. You may take some notes now, which you must not use during your talk. Do you have any questions?"

## The TSST Chairperson of the Panel

**Opening up the session**

“Please step behind the line and begin your talk".

If the participant talks for longer than 3 minutes, then interrupt.

If participant stops before 3 minutes, wait for about 20s and then say: “You still have time”. If participant does not continue, start asking questions.

**Questions to ask the research participant during the “job interview”**

(After 3 minutes of free speech)

- Why do you think you are especially well-qualified for this task?
- Why do you think you are better qualified then the other applicants?
- What do your family/friends especially appreciate about you?
- What do you appreciate about your friends?
- What do you appreciate about colleagues?
- You just pointed out that you were especially good at…, what other characteristics qualify you?
- You just mentioned you qualities in respect to…, what do you in particular think about…?
- You just spoke about…, what exactly do you then think about…?
- Please complete the following sentence: “I am the best at/in…”
- Please list your strengths!
- Please list your weaknesses!
- What kind of leading qualities do you have?
- What do you think about teamwork?
- Where do you see your position in a team?
- What can you constructively add to a team?
- You just mentioned that you really appreciate teamwork, what do you think about lone fighters?
- What do you think about job interviews?
- What do your employees appreciate about you most?
- Would you be willing to work overtime without compensation?
- Would you be willing to work on the weekends if this be deemed necessary?
- What kind of leading qualities do you expect from your employees?
- What kind of qualities to you expect from your co-workers?
- Under what circumstances would you be willing to compensate for the mistakes your co-workers make?
- Would you lie in order to gain an advantage?
- What do you think about the saying “Everybody determines his own luck”

**Arithmetic task**

“We now want you to solve a calculation task. Please count aloud backwards from 2023 in steps of 17. Please calculate as quickly and correctly as possible. Should you miscalculate, we will point out your mistake and you have to start all over again. Do you have any questions?"

| 2023 | 1683 | 1343 | 1003 | 663 | 323 |
| --- | --- | --- | --- | --- | --- |
| 2006 | 1666 | 1326 | 986 | 646 | 306 |
| 1989 | 1649 | 1309 | 969 | 629 | 289 |
| 1972 | 1632 | 1292 | 952 | 612 | 272 |
| 1955 | 1615 | 1275 | 935 | 595 | 255 |
| 1938 | 1598 | 1258 | 918 | 578 | 238 |
| 1921 | 1581 | 1241 | 901 | 561 | 221 |
| 1904 | 1564 | 1224 | 884 | 544 | 204 |
| 1887 | 1547 | 1207 | 867 | 527 | 187 |
| 1870 | 1530 | 1190 | 850 | 510 | 170 |
| 1853 | 1513 | 1173 | 833 | 493 | 153 |
| 1836 | 1496 | 1156 | 816 | 476 | 136 |
| 1819 | 1479 | 1139 | 799 | 459 | 119 |
| 1802 | 1462 | 1122 | 782 | 442 | 102 |
| 1785 | 1445 | 1105 | 765 | 425 | 85 |
| 1768 | 1428 | 1088 | 748 | 408 | 68 |
| 1751 | 1411 | 1071 | 731 | 391 | 51 |
| 1734 | 1394 | 1054 | 714 | 374 | 34 |
| 1717 | 1377 | 1037 | 697 | 357 | 17 |
| 1700 | 1360 | 1020 | 680 | 340 | 0 |

**Should the participant miscalculate?**

"Error. 2023."
